# 3D measurement techniques for the hindfoot alignment angle from weight-bearing CT in a clinical population

**DOI:** 10.1038/s41598-022-21440-9

**Published:** 2022-10-07

**Authors:** Chiara Pavani, Claudio Belvedere, Maurizio Ortolani, Mauro Girolami, Stefano Durante, Lisa Berti, Alberto Leardini

**Affiliations:** 1 Movement Analysis Laboratory, IRCCS Istituto Ortopedico Rizzoli, Via di Barbiano 1/10, Bologna, Italy; 2 Bentivoglio Orthopaedic Ward, IRCCS Istituto Ortopedico Rizzoli, Bologna, Italy; 3 Management of Health Professions IRCCS S. Orsola-Malpighi Hospital, Bologna, Italy

**Keywords:** Biomedical engineering, Medical research

## Abstract

Cone-beam CT (CBCT) scans now enable accurate measurements on foot skeletal structures with the advantage of observing these in 3D and in weight-bearing. Among the most common skeletal deformities, the varus/valgus of the hindfoot is the most complex to be represented, and a number of measure proposals have been published. This study aims to analyze and to compare these measurements from CBCT scans in a real clinical population with large such deformity. Ten patients with severe acquired adult flatfoot and indication for surgery underwent CBCT scans (Carestream, USA) while standing on that leg, before and after surgical correction. Corresponding 3D shape of each bone of the distal shank and hindfoot were defined (Materialise, Belgium). Six different techniques from the literature were used to calculate the varus/valgus deformity, i.e. the inclination of the hindfoot in the frontal plane of the shank. Standard clinical measurements by goniometers were taken for comparison. According to these techniques, and starting from a careful 3D reconstruction of the relevant foot skeletal structures, a large spectrum of measurements was found to represent the same hindfoot alignment angle. Most of them were very different from the traditional clinical measures. The assessment of the pre-operative valgus deformity and of the corresponding post-operative correction varied considerably. CBCT finally allows 3D assessment of foot deformities in weight-bearing. Measurements from the different available techniques do not compare well, as they are based on very different approaches. It is recommended to be aware of the anatomical and functional concepts behind these techniques before clinical and surgical conclusions.

## Introduction

Acquired adult flatfoot, more recently defined as ‘progressive collapsing foot deformity’^1^, implies multiple, complex and combined three-dimensional (3D) deformities, including flattening of the medial longitudinal arch, abduction of the forefoot and, in particular, valgus of the hindfoot^1–4^. In support to the standard clinical assessments^1,5^, accurate measurements on foot bones should be established^6–11^ to evaluate the severity of the deformities, to plan for possible correction procedures and to assess the outcomes. Unfortunately, simple foot bone alignments in weight-bearing are usually assessed based on X-ray pictures with the subject in upright postures^6,12^, thus missing essential 3D observations and suffering of bones’ superimposition and reproducibility, particularly for the analysis of the subtalar joint^13^. Nowadays, 3D scans of the foot in weight-bearing conditions are possible using the cone beam technology (CBCT)^7,14^. These modern devices provide valuable 3D scans of the foot and ankle with subjects in one- or two-leg stance^15–19^, with low radiation doses, high spatial resolution, convenient ergonomy and suitable post-processing^7,20–24^. CBCT scans are proving to be very valuable in many different clinical contexts^1,5,7,12,14–18,21–23,25,26^, but the assessment of hindfoot alignment angle (HAA) in severely deformed feet can be among the areas of most considerable benefit.

Starting from CBCT scans, quantitative analysis of the foot’s overall skeletal geometry requires 3D reconstruction of the bone external shape, i.e. segmentation^27^, in the final format of digital mesh. Bone alignments and orientation angles are calculated by reference frames or anatomical axes embedded into these 3D bone models. This finally overcomes the traditional bi-dimensional angular measurements from radiographs^6,26^ and enables more comprehensive representations of foot bone alignments in 3D^6,8,9^. 3D measurements from CBCT are no longer affected by manual digitisation and confounding projections associated with foot malposition or severe deformities. Another advantage of the CBCT-based analyses is the weight-bearing condition, as demonstrated recently in flatfoot patients^14^. 3D measurements of hindfoot alignments in weight-bearing added considerably to standard clinical evaluations^28^ and showed that traditional bi-dimensional measurements from radiographs significantly underestimated the severity of the flatfoot deformity^29–31^.

HAA, also called the varus/valgus of the calcaneus or the heel valgus or the hindfoot coronal alignment, has been largely used to characterize deformities flatfoot patients. HAA should be measured in weight-bearing, i.e. in single- or double-leg upright posture^25^. Classical assessments in this condition were based on standard goniometers^32,33^, but this was shown to be unreliable^34^, frequently underestimating the valgus deformity when compared to radiographical assessments^35^. A laser-assisted measure has been recently proposed^36^, but this is still based on a qualitative observation of the external aspect of the back of the ankle. HAA has been measured largely on planar X-ray pictures^37–39^, mostly using the hindfoot alignment view^34,40–43^ or the long axial view^44^. These can be affected by errors due to malpositioning of the foot and superimposition of its bones. A new classification system apparently has improved inter-observer reliability^45^.

A more robust assessment of the HAA is now possible by using CBCT devices^46^, potentially able to support original representations of the complex flatfoot deformities in 3D^28,47^. However, a number of different methods have been proposed^28,48^, mostly by considering or projecting in the frontal plane the longitudinal axis of the tibia and the vertical axis of the calcaneus. The former is the mid-diaphyseal axis, usually obtained by interpolating midpoints of the tibial external shape, whereas the latter requires more sophisticated methods. The vertical axis of the calcaneus was in fact defined over its medial or lateral osseous contours^30,43,49,50^, or by the upper and lower profiles^39^. A more complete representation of hindfoot orientation would also imply an axis between the most inferior point of the calcaneus and a point on the talus, thus defining a talocalcaneal inclination^51^. Measurements of the HAA can be very different also from what it is assessed clinically by external observations. For this reason, it has been recommended to target the calcaneal posterior tuberosity when using radiographs^48^. These methods can definitely benefit of 3D bone models of patients from CBCT.

The scope of the present work is to assess a number of different techniques from the literature for HAA measure from CBCT scans. To appreciate and understand better the differences, these techniques were exploited in a population of flatfoot patients, taken before and after a surgical correction. These measurements were also compared with those using more traditional techniques, i.e. based on the external aspect of the distal shank and the hindfoot, taken originally from the same CBCT scans. The hypothesis is that these techniques result in largely different values of hindfoot alignment, most of which different also from the traditional clinical measures.

## Material and methods

The different techniques for HAA measure from CBCT scans are reported with some details here below. Before this, the clinical population used for this preliminary comparison and the collection and reconstruction procedures for this anatomical data set are reported. This population was selected to represent well large hindfoot deformities and their expected corrections.

### Clinical population, surgery, ethics, and CBCT data collection

Ten patients (4 male/6 female, 52.6 ± 15.2 years old, 79.5 ± 13.3 kg weight, 168.2 ± 7.7 cm height, 28.1 ± 4.1 BMI) with indication for surgical correction were analyzed before operation and at about 6 months follow-up. The Mini Bone Block Distraction Subtalar Arthrodesis^52,53^ technique was performed, which entails the insertion of a corticocancellous bone autograft, harvested from the ipsilateral proximal tibia, to be positioned and pressed into the sinus tarsi to enhance fusion between the talus and calcaneus.

The affected foot (4 right/6 left) was scanned in single-leg upright posture, with a CBCT device (OnSight 3D Extremity System, Carestream, Rochester, NY—more technical details in Ortolani et al.^9^). The present study received internal review board approval. The investigation was performed in accordance with the relevant guidelines and regulations. Informed consent to participation in this study and to publish relevant anonymized information and images was obtained by all patients. All patients were above the age of 18 years.

### 3D bone model reconstruction

**Figure 1:**
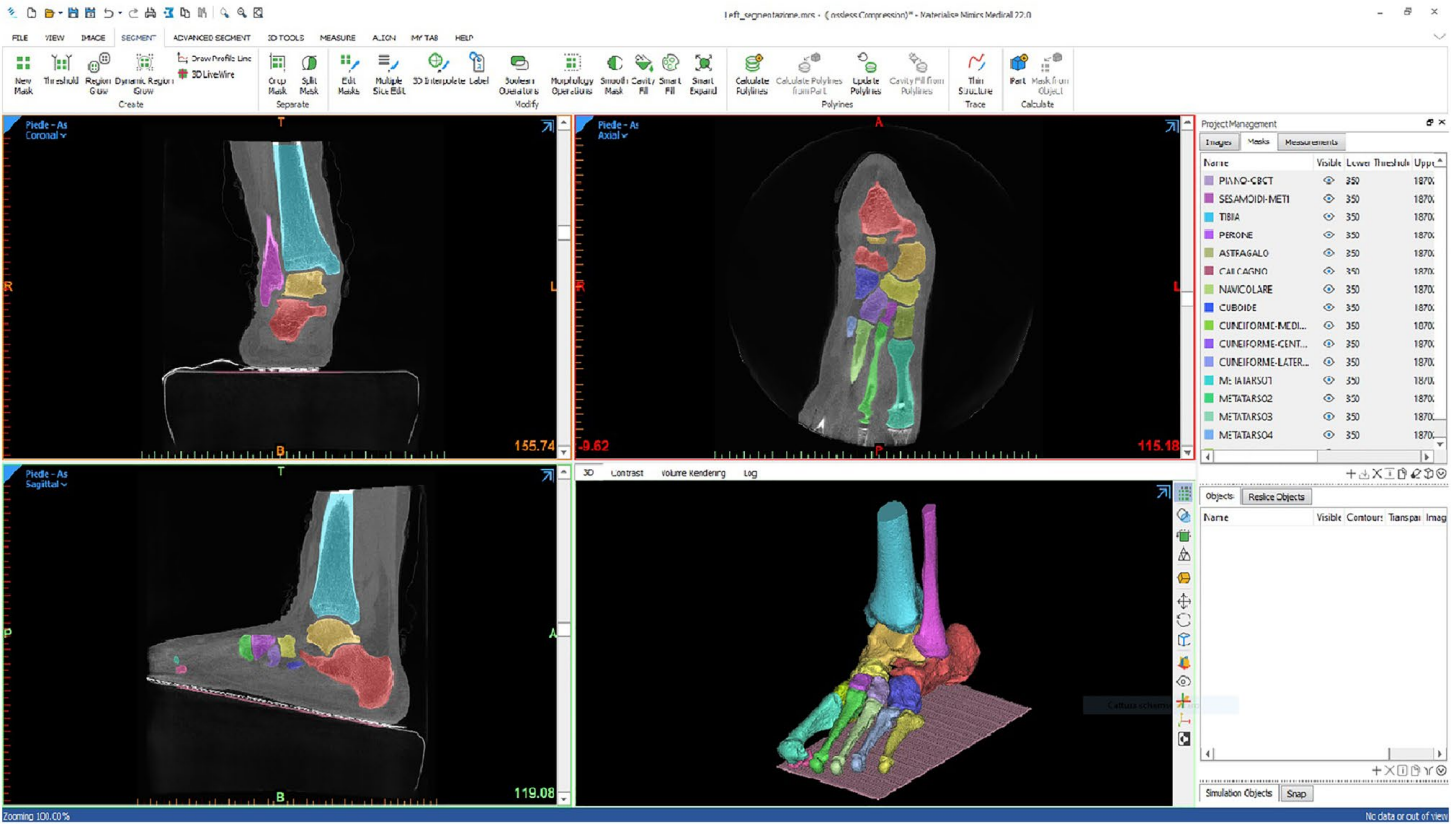
A screenshot from the 3D reconstruction of the foot bones from a CBCT scan by Mimics Innovation Suite (Materialise, Belgium) software. Each bone segment has a different colour, in both the 3D view (bottom-right) and in the three anatomical planar views; in this antero-posterior slice (top-left), the tibia, fibula, calcaneus and talus bone segments are depicted. In the sagittal view (bottom-left), apparently the foot is an inclined ground, but this is accounted for to the inclination of the gantry in the present device, i.e. the foot is on the horizontal ground.

For each foot scan, 960 CT images at 0.26 mm distance were exported in a Dicom file. This was processed in Mimics Innovation Suite version 22.0 (Materialise, Leuven, Belgium) by a single operator to work out a 3D mesh for distal tibia and fibula and the foot bones (Fig. 1, bottom-right), i.e. segmentation. This entails the semi-automatic identification separately of each foot bone, starting from the three anatomical views in the Dicom file. The ground under the foot was segmented as well, and taken in the overall 3D model file. This was assumed as the global transverse plane, which was necessary for the calculation of the absolute orientation of the foot bones. Because of the surgical procedure, the corticocancellous autograft was found between the talus and calcaneus in the Dicom files of the post-operative CBCT scans; this resulted in a more difficult segmentation of these two bones, but did not affect their final 3D pose estimation.

### Calculation of anatomical planes and bone axes

**Figure 2:**
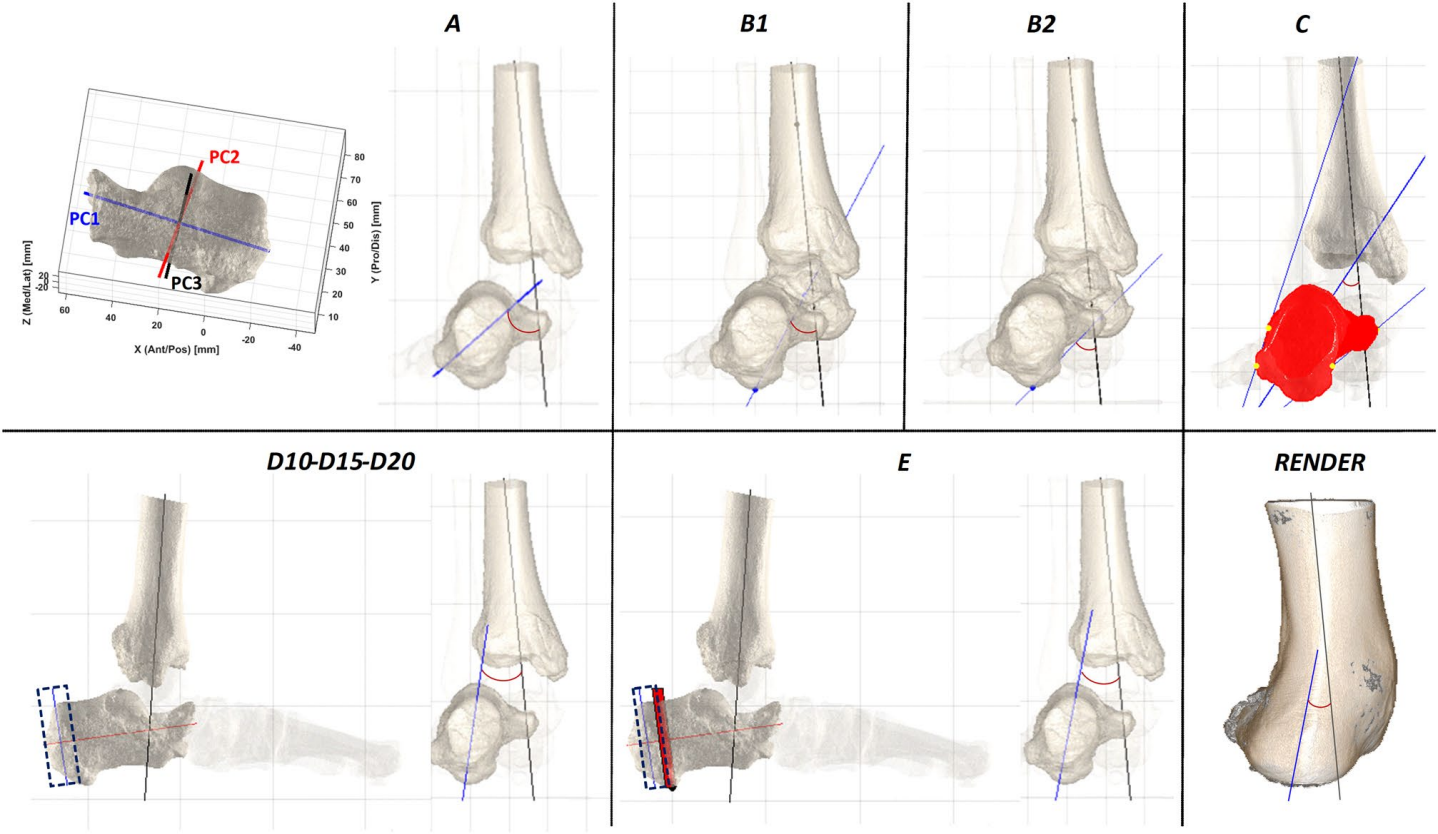
Representation of all the techniques for HAA calculation. In the frontal view, the longitudinal axis of the tibial distal diaphysis (black) and the hindfoot vertical axis (blue) are shown for each technique. For technique A, the three principal component axes of the PCA applied to the calcaneus are also shown. For techniques D and E, the sagittal views are also to better represent the posterior part of the calcaneus. Lastly, the bottom-right (RENDER) is the posterior view of the CBCT rendering of the same scan.

These files, in STL format, were imported in Matlab (Mathworks Inc., Natick, MA, USA) to be processed according to the following six different techniques for the calculation of the varus/valgus of the hindfoot, i.e. the HAA (Fig. 2).

Before these calculations, an anatomical reference frame was defined for the entire foot (FootAF), with the dorsi/plantar axis orthogonal to the ground plane, the antero/posterior axis on the ground joining the projections of the most plantar points of the calcaneus and second metatarsal head, and the medio/lateral axis orthogonal to these two. All bone models were then realigned in this FootAF.

For all of the following techniques, HAA was taken as the angle between the projections of the vertical axes of the hindfoot and of the tibia on the FootAF frontal plane. For the tibia, the available diaphyseal section of the bone was used for the calculation of a relevant anatomical frame based on the Principal Component Analysis (PCA)^8^. This technique entails the calculation of three orthogonal axes in one shot, those with the highest variance of the 3D coordinates of the bone surface points. These three axes are thus assumed to be the antero-posterior, medio-lateral and vertical anatomical axes of that section of the bone.

The six different techniques for the calculation of the HAA are defined as follows (Fig. 2):

A. The PCA-based anatomical reference frame^8^ was defined also for the calcaneus. Its vertical, i.e. the dorsi-plantar, axis was projected onto the frontal plane of the FootAF and the angle between this projection and that of the tibial vertical axis was taken as the HAA.
B1. A talo-calcaneal axis was defined by connecting the most plantar point of the calcaneus to the centroid of the talus. As above, this axis was projected onto the frontal plane of the FootAF for the HAA angle to be calculated with the equivalent from the tibia.
B1. A definition very similar to B1 takes the same most plantar point of the calcaneus and the centre of the middle facet of the talus, endorsed as the centre of the subtalar joint^54^.
C. Inspired by the approach in Williamson et al.^43^, the line segments representing lateral and medial contours of the calcaneus, once projected onto the frontal plane of the FootAF, were thus searched. More precisely, the full silhouette of this projection was divided in the top and the bottom part. In each of these two parts, the most lateral and the most medial points were identified, and these lateral and medial line segments were thus defined. The HAA was the angle between the bisector of these two line segments and the vertical axis projection of the tibia.
D. To figure out the inclination of the sole posterior aspect of the calcaneus, that somehow observed at the external aspect of the rearfoot, this part of the bone was encapsulated in a 3D box, with one side orthogonal to the longitudinal axis of this bone (Fig. 2, D10–D15–D20). From the most posterior point of the calcaneus, three boxes encapsulating 10%, 15%, and 20% of the length of the longitudinal line segment of the calcaneus, respectively, were defined. The PCA technique was applied to each of these three bone sections of the calcaneus contained in these three boxes, and the resulting vertical axis was projected onto the frontal plane for the calculation of three HAAs, i.e. D10, D15 and D20.
E. With the same approach of D, a single 3D box, still with one side orthogonal to the longitudinal axis of the calcaneus, was defined to encapsulate its posterior aspect. Starting from the most posterior point, the width of the box was defined by progressing anteriorly until the most plantar point of the calcaneus is encapsulated. As in D, the PCA technique was then applied to this posterior section of the calcaneus to obtain the vertical axis of its posterior part, for the calculation of the HAA.

Techniques D and E still need to define first a reference frame for the entire calcaneus, in particular for the definition of the longitudinal axis and the dorsi-plantar direction, and this was still based on the PCA technique.

For comparison, traditional clinical measurements of HAA were taken on these feet on the same day with a goniometer^33^ by a single expert operator. For a possibly more consistent comparison between external measurements, i.e. based on the full body segment, and internal measurements, i.e. based on inner bones, the HAA was taken by a goniometer also from a posterior view of the 3D rendering of the original CBCT scans after adjusting the contrast to depict the skin surfaces (Fig. 2, RENDER). These images were printed out, and the HAA was taken by five physicians familiar with these measures. All of these measurements were taken pre-op and post-op.

The Student t-test was used to analyse the statistically significant difference of the measurements from these techniques, i.e. *p* values < 0.05, and also incidentally the ability of these techniques to reveal differences between pre-op and post-op condition.

### Ethical approval

The study was approved by the ethical committee of the IRCCS Istituto Ortopedico Rizzoli, Bologna – Italy (Prot. Gen 0012502, November 5, 2018). The authors certify that the institution approved the investigation protocol and that all investigations were conducted in compliance with ethical standard of research. Specifically, the investigation methodology was performed in accordance with the relevant guidelines and regulations. Informed consent for participation in this study and to publish related anonymized information/images was obtained from all patients. All patients were above the age of 18 years.

### Informed consent

Signed informed consent for participation in this study and to publish related anonymized information/images was obtained from all patients. All patients were above the age of 18 years.

## Results

**Figure 3:**
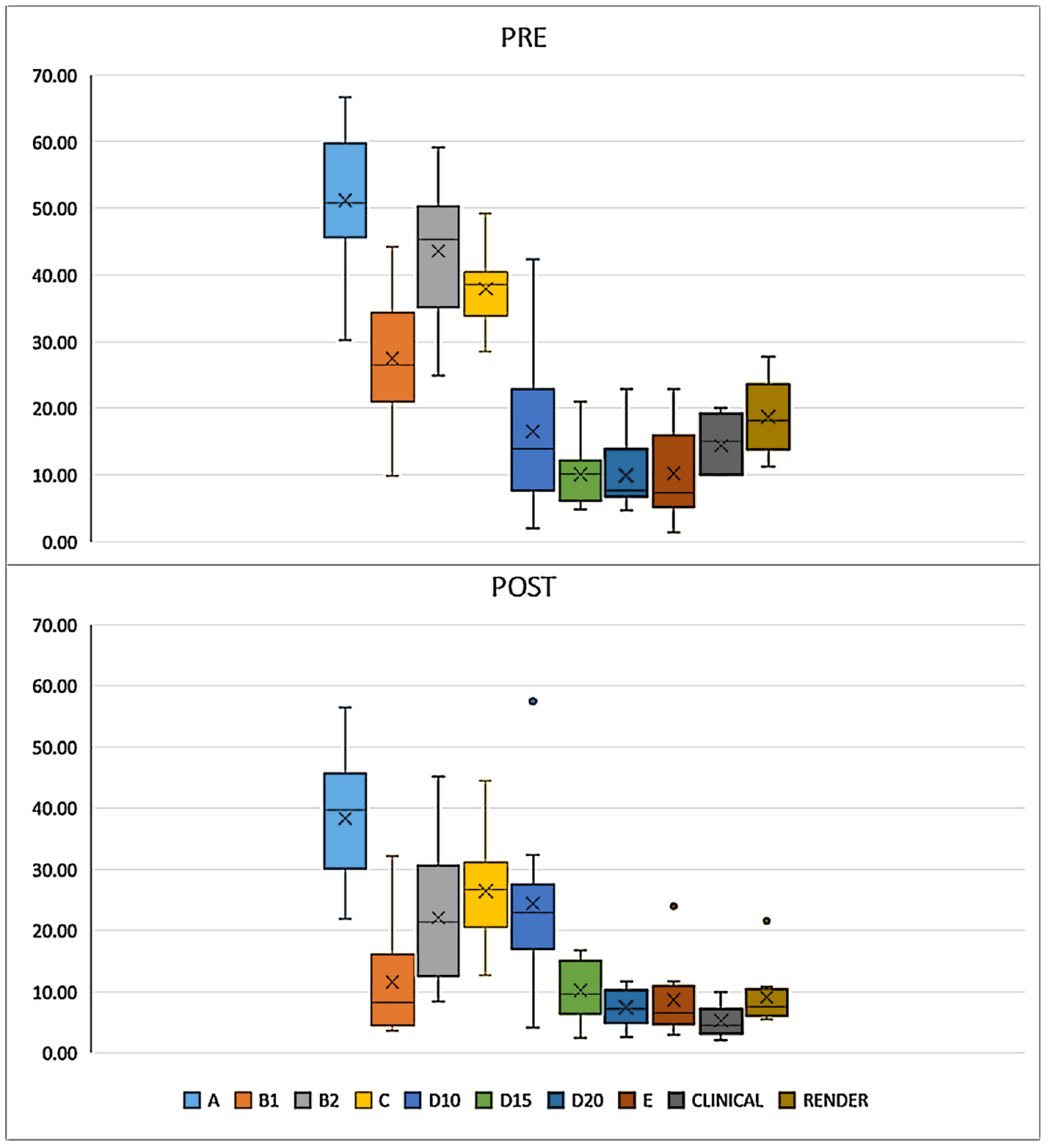
Boxplot with relevant outliers; the HAA measurements from all techniques for pre-op (top) and post-op (bottom). Outliers were not found in the pre-op values.

A large spectrum of measurements was found using the six different techniques on the same foot scans, with mean HAA values pre-op over the 10 patients from 51° (technique A) to less than 10° (technique D20) (Table 1, Fig. 3), easily accounted for by the very different anatomical and geometrical approaches. The best mean-to-STD ratio was observed for technique C, thus supporting a better ability for consistent assessments, at least in the present clinical population. Most of these techniques, based on careful 3D bone model reconstruction and geometrical analyses, showed HAA values very different from those obtained with the traditional clinical measurements based on a simple goniometer. As expected, these traditional measurements are similar to CBCT-based techniques focused on the posterior aspect of the calcaneus (D10 D15 D20), which is somehow implied in these clinical measures. This is particularly true for the measurements on the hindfoot rendering (WBCT RENDER), which were taken in the same exact loading conditions as the CBCT-based measurements. Both PCA-based techniques (A) and those based on the overall hindfoot (B1 B2) overestimate the HAA with respect to all the other techniques.

**Table 1:** Mean and standard deviation (STD) of the HAA measurements (unit is degrees) over all the patients for each of the different CBCT-based techniques and for the two based on the external aspect and a goniometer (CLINICAL, RENDER).

	PRE		POST		*p* value
	Mean	STD	Mean	STD	
A	51.19	10.27	38.32	10.42	0.0052
B1	27.49	10.09	11.53	8.90	0.0005
B2	43.58	10.10	22.10	11.15	0.0002
C	37.94	5.73	26.38	8.99	0.0027
D10	16.49	12.81	24.40	13.92	0.0089
D15	10.06	4.86	10.21	4.93	**0.9408**
D20	9.92	5.63	7.42	3.03	**0.2904**
E	10.26	6.74	8.64	6.13	**0.5719**
CLINICAL	14.40	4.25	5.20	2.53	0.0000
RENDER	18.70	5.74	9.08	4.85	0.0001

This is reported for the pre-op and the post-op condition, along with the *p* values for the difference between these two conditions (values not statistically significant in bold).

Significance of the difference between the techniques was found inconsistent with the present measurements taken pre- and post-op (Table 2). As expected, technique A based on the PCA algorithms provides values very different from all other more anatomical based techniques, both pre- and posto-op. In pre-op conditions, when the deformity is still severe, technique D10 results in values not significantly different from all those techniques based on observations of the back of the calcaneus, either from CBCT of by goniometer, and thus it can represent them well. In post-op conditions, this role seems to be played by B1. Interestingly, B1 and B2 performed very differently post-op, this pointing out the role of the centre of the subtalar joint. We found significance differences between the techniques in 10 out of the 45 comparisons pre-op, 14 post-op; i.e. with smaller deformity these are more consistent.

**Table 2:** *p* values for the statistically significant differences between the techniques (values not statistically significant in bold).

	A	B1	B2	C	D10	D15	D20	E	Clinical	Render
**PRE**										
A										
B1	0.0000									
B2	0.0082	0.0000								
C	0.0014	0.0033	**0.0687**							
D10	0.0001	**0.0718**	0.0007	0.0024						
D15	0.0000	0.0001	0.0000	0.0000	**0.1067**					
D20	0.0000	0.0000	0.0000	0.0000	**0.1841**	**0.9008**				
E	0.0000	0.0001	0.0000	0.0000	**0.236**	**0.9059**	**0.7615**			
Clinical	0.0000	0.0016	0.0000	0.0000	**0.6536**	0.0274	0.0148	0.0318		
Render	0.0000	0.0043	0.0000	0.0000	**0.6581**	0.0012	0.0001	0.0004	0.0008	
**POST**										
A										
B1	0.0000									
B2	0.0002	0.0000								
C	0.0116	0.0001	**0.1626**							
D10	0.035	0.0407	**0.6917**	**0.7322**						
D15	0.0001	**0.7239**	0.0172	0.0014	0.0022					
D20	0.0000	**0.2104**	0.0035	0.0002	0.004	**0.0686**				
E	0.0000	**0.4526**	0.0151	0.0023	0.0091	**0.5366**	**0.4609**			
Clinical	0.0000	0.0431	0.0008	0.0000	0.0025	0.0233	0.0248	**0.0639**		
Render	0.0000	**0.242**	0.0008	0.0001	0.0159	**0.6658**	0.**3257**	**0.8492**	0.0068	

Incidentally, the present calculations on severe flatfeet using these different techniques revealed considerable corrections of the pre-op valgus deformity of the hindfoot (Table 1, Fig. 3). After operation, the variability over the patients was found smaller, accounted for by the precise surgical goal. The large differences between the various techniques would have affected in case also the resulting degree of surgical correction, statistically significant differences pre-op vs post-op being found for all the techniques except D15, D20 and E.

## Discussion

A large hindfoot valgus, i.e. HAA, along with the forefoot abduction, and the collapse of the medial longitudinal arch, is a major deformity of flatfoot. It has been shown that, when analysing the different components of the deformity separately, hindfoot valgus was found in most of the cases^45^. A thorough assessment of the HAA is thus essential for proper diagnosis and for the assessment of treatments for these patients. The measure of this angle has been largely performed using standard goniometers and radiographs to assess the external appearance and the bone orientation, respectively. The former is hampered by subjective identification of the axes and the latter by the superimposition of the bones. More accurate analyses of HAAs in weight-bearing are now made possible by CBCT devices^46^, which definitely have the potential to show the complex and critical three-planar deformities of the entire flatfoot in a condition more similar to the daily living activities^14,28,47^. By taking advantage of this modern technology, a number of techniques have been reported in the literature for calculating the HAA from CT scans^28,30,39,43,48–51^. The longitudinal axis of the tibia, or shank, as well as the vertical axis of the calcaneus can be represented, calculated and projected in many different ways. The present work wants to quantify the differences between these techniques, in a real clinical population, i.e. flatfoot patients before and after a known surgical correction.

The shape of the bones of the distal shank and the hindfoot are complex, and even when a careful 3D model is available, defining and calculating the relevant anatomical axes is always problematic and thus disputed. This is particularly true when semi-automatic procedures should be established to limit subjective assessments of the HAA. Nevertheless, for the first time, HAA measurements were taken on 3D bone models, thus overcoming the known drawbacks of conventional bidimensional radiographs. This study shows the extent to which HAA values can be very different even when an accurate 3D model of the relevant bones is available. These digital models can be projected and handled easily for the most appropriate representations of the axes necessary for the calculation of the HAA. In fact, many portions and landmarks of the bones can be considered, which resulted here in different values, as also pointed out recently elsewhere^28^. In the present work, the HAA measurements based on CBCT scans were obtained in a repeatable way, as the relevant procedures were operator-independent. These values were compared also to traditional manual measurements, i.e. with a goniometer, necessarily operator-dependent. The latter were taken to provide a reference to current, largely more frequent, semi-quantitative assessments of hindfoot deformity. The present findings show that all CT scan based measures differ considerably from the routine clinical evaluations, both before and after a surgical treatment (Tables 1 and 2). The present work however cannot establish a single best technique, which should be chosen according to the scopes and the anatomical target of the measures. It is also reiterated here that clinical measurements with a goniometer have a large inter-rated variability.

The HAA values obtained from CBCT scans by using the proposed techniques were found to be very different (Table 1 and 2). The first technique (A) shows the highest mean values and a very high STD. This can be accounted for by the automatic calculations by the PCA technique, which identifies the three anatomical axes, including the relevant vertical, over the whole calcaneus bone model; by looking at three main directions in a typical mesh, the longitudinal one has likely the largest variance, and the vertical, researched herein, has the second largest. The second technique (B) also takes into account the talus, this being a relevant structure of the hindfoot, well representative of the deformity, and also directly involved in the surgical correction. Relevant values using B1 are about half of those from technique A. A slightly different technique, B2 aimed to target the centre of the subtalar joint mobility. This was recently identified as a peculiar landmark^54^, anatomically identified at the centre of the middle facet of the talus, thus easy to depict in 3D bone models. The results of this further HAA calculation do differ considerably from B1, accounted for by the anatomical position of this landmark. Smaller STDs were observed with technique C, inspired by the Williamson et al.^43^ approach, thus suggesting that a careful definition of a medial and lateral contour of the calcaneus results in a proper repeatable calculation of its vertical axis. Unlike that original paper, with calcaneus inclination measurements based on manual definition of medial and lateral line segments from X-rays pictures, the present calculations rely on accurate 3D reconstructions of this bone, so that the identification of these contours are based on careful projections of isolated calcaneal surfaces, thus unhampered by the superimposition of other bones. Besides these considerations of the morphology and inclination of the whole calcaneus, a focus on only the posterior part of this bone was also investigated (techniques D and E) to determine its vertical axis, possibly to better mimic the clinical measure of the HAA^28^. Starting from the whole 3D bone model, different portions of the back of the calcaneus were isolated (D10 D15 D20), and the PCA was applied to these portions in the search of the vertical axis as the one having the largest variance. D15 and D20 techniques resulted in the lowest mean values over all the patients (Table 1), which demonstrates that the posterior tuberosity is oriented less in valgus than the rest of the calcaneus. However, among the three percentages of these posterior portions, only 20%, i.e. D20, seems to represent well the correction in varus obtained by surgery, and this was found consistent with the values based on the plantar point (technique E). Further improvements of techniques D and E will imply a thorough definition of the posterior portion of the calcaneus, to be isolated and to be used for a possible more reliable and repeatable calculation of the HAA.

The present results also contribute to the comparison of 3D radiographical, i.e. CBCT, with clinical measurements of the HAA, both in weight-bearing. In the present example population, pre-op and post-op, the clinical values were considerably smaller than the CBCT based values, which would indicate a sort of smoothing and correcting effect of the soft tissues surrounding the calcaneus bone. In this respect, by using CBCT images in weight-bearing and measuring the HAA by looking at the bone models or at the external, i.e. cutaneous, profiles of the shank and hindfoot, Burssens et al.^46^ showed that the latter measurements were larger with respect to the former. On the other hand, de Cesar Netto et al.^28^ showed that the HAA measured on 3D bone models was higher than corresponding clinical measurements, these being taken directly on the patients and also from 3D rendering from CBCT scans. In the present study, according to both CBCT and clinical measurements of the HAA, pre-op hindfoot misalignment was considerably corrected after surgery. The remaining valgus was consistent with a physiological joint and, in any case, targeted by the surgeons and the surgical procedure.

The presents study is limited by the small number of patients analyzed; however, their feet represent well a target population for the use of the techniques here investigated. In addition, the present post-operative condition have addressed the potentially critical presence of bone grafts in the CBCT scans. A concern, in fact, was associated with the segmentation of the talus and calcaneus after surgery because the insertion of the tibial bone graft makes the separate identification of the two bones more difficult. Eventually, their segmentation was carried out by superimposing, with a 3D best-fit procedure, the corresponding bone models from the pre-op scans to the good quality portions of the post-op models. This resulted in a slightly more complicated segmentation process but definitely did not affect the following steps for HAA calculations. This is not common in hindfoot surgery, though arthrodesis and arthroereisis do imply similar problems. Other populations of feet, such as patients with osteoarthritis or osteoporosis, as well as normal subjects, may reveal slightly differences among the techniques in the future. Another relevant limitation is the portion of the distal shank here analyzed. The HAA in fact is typically measured on lower leg radiographs, where the full tibia and fibula are shown, whereas the field-of-view of the present CBCT device is much shorter. In this study, about one third of the tibial diaphysis was available, and the relevant 3D bone model was enough to define a reliable longitudinal axis for this bone. In any case, this axis was taken for all the techniques so that the HAA differences are accounted for only by the vertical axis of the calcaneus, as desired. It has also been shown that HAA calculation can be robust even with a short portion of the tibia only^46^. Finally, only one expert operator run the bone segmentations and the HAA calculations; however both of these procedures have a very high degree of automation^9^, and thus very little differences are expected from different operators.

In conclusion, the values of HAA obtained from the techniques proposed in the literature and here analyzed in a clinical population differ considerably, likely because of the different original aims, adopted techniques, and exact targets. Among these techniques, those targeting the posterior portion of the calcaneus showed measurements similar to most of the traditional clinical measures, based in fact on the external posterior aspect of the hindfoot. Relevant radiological and clinical studies and also single-patient observations must be aware of what exactly is calculated in each of these techniques. For cautious assessments of bone and joint alignments, calculations should be based on weight-bearing CBCT scans and thorough 3D geometric analyses, as in the present work.

## Data Availability

The datasets used and/or analyzed during the current study are available from the corresponding author upon reasonable request.
