# Author Correction: 3D measurement techniques for the hindfoot alignment angle from weight-bearing CT in a clinical population

**DOI:** 10.1038/s41598-022-24322-2

**Published:** 2022-11-18

**Authors:** Chiara Pavani, Claudio Belvedere, Maurizio Ortolani, Mauro Girolami, Stefano Durante, Lisa Berti, Alberto Leardini

**Affiliations:** 1 Movement Analysis Laboratory, IRCCS Istituto Ortopedico Rizzoli, Via di Barbiano 1/10, Bologna, Italy; 2 Bentivoglio Orthopaedic Ward, IRCCS Istituto Ortopedico Rizzoli, Bologna, Italy; 3 Management of Health Professions IRCCS S. Orsola-Malpighi Hospital, Bologna, Italy

Correction to: *Scientific Reports* 10.1038/s41598-022-21440-9, published online 07 October 2022

The original version of this Article contained an error in the Funding section.

“This study was partially funded by the Italian Ministry of Health’s “5 Per Mille” program.”

now reads:

“This study was partially funded by the Italian Ministry of Health under the “5 per mille” program.”

The original Article has been corrected.

